# TcOPT3, a Member of Oligopeptide Transporters from the Hyperaccumulator *Thlaspi caerulescens*, Is a Novel Fe/Zn/Cd/Cu Transporter

**DOI:** 10.1371/journal.pone.0038535

**Published:** 2012-06-22

**Authors:** Yi Ting Hu, Feng Ming, Wei Wei Chen, Jing Ying Yan, Zheng Yu Xu, Gui Xin Li, Chun Yan Xu, Jian Li Yang, Shao Jian Zheng

**Affiliations:** 1 College of Environmental and Resource Sciences, Zhejiang University, Hanzhou, China; 2 Key Laboratory of Conservation Biology for Endangered Wildlife, Ministry of Education, College of Life Science, Zhejiang University, Hanzhou, China; 3 Institute of Plant Biology, School of Life Science, Fudan University, Shanghai, China; 4 The Anhui Provincial Lab of Nutrient Cycling, Resources and Environment; Institute of Soil and Fertilizer, Anhui Academy of Agricultural Sciences, Hefei, China; 5 College of Agronomy and Biotechnology, Zhejiang University, Hanzhou, China; 6 State Environmental Protection Administration of Radiation Environmental Monitoring Technology Center, Hanghzou, China; Kansas State University, United States of America

## Abstract

**Background:**

*Thlaspi caerulescens* is a natural selected heavy metal hyperaccumulator that can not only tolerate but also accumulate extremely high levels of heavy metals in the shoots. Thus, to identify the transportors involved in metal long-distance transportation is very important for understanding the mechanism of heavy metal accumulation in this hyperaccumulator.

**Methodology/Principal Findings:**

We cloned and characterized a novel gene *TcOPT3* of OPT family from *T. caerulescens*. *TcOPT3* was pronouncedly expressed in aerial parts, including stem and leaf. Moreover, *in situ* hybridization analyses showed that *TcOPT3* expressed in the plant vascular systems, especially in the pericycle cells that may be involved in the long-distance transportation. The expression of *TcOPT3* was highly induced by iron (Fe) and zinc (Zn) deficiency, especially in the stem and leaf. Sub-cellular localization showed that TcOPT3 was a plasma membrane-localized protein. Furthermore, heterogonous expression of TcOPT3 by mutant yeast (*Saccharomyces cerevisiae*) complementation experiments demonstrated that *TcOPT3* could transport Fe^2+^ and Zn^2+^. Moreover, expression of TcOPT3 in yeast increased metal (Fe, Zn, Cu and Cd) accumulation and resulted in an increased sensitivity to cadmium (Cd) and copper (Cu).

**Conclusions:**

Our data demonstrated that *TcOPT3* might encode an Fe/Zn/Cd/Cu influx transporter with broad-substrate. This is the first report showing that TcOPT3 may be involved in metal long-distance transportation and contribute to the heavy metal hyperaccumulation.

## Introduction

Metal hyperaccumulators can not only tolerate high concentration of heavy metals in the soils but also take them up actively and accumulate and distribute them to appropriate tissues at extreme high levels, thus make them very attractive for the remediation of heavy metal polluted soils [1]. A large amount of different metal hyperaccumulators have been recognized in different regions all over the world [2], among them, *Thlaspi caerulescens,* a Cd/Zn/Ni hyperaccumulator, has been used as a model plant to study the physiological and molecular mechanisms of heavy metal hyperaccumulation [3], [4].

The efficient metal loading and unloading ability in the vascular systems was regarded as the key step of hyperaccumulating process for hyperaccumulators [5]. Nonetheless, knowledge about the mechanisms and proteins involved in transporting heavy metals from soil via the roots and stems into their storage sites is much limited. Transporter families, including ZIP (ZRT/IRT like Protein), Nramp (Natural Resistance and Macrophage Protein), MTP (Metal Tolerance Protein), HMA (Heavy Metal transporting P-type ATPase), ABC-type (ATP-binding cassette), COPT family CTR/COPT (high-affinity Cu transporters) as well as YSL (Yellow strip1-Like transporters) families have been porved to involve in transit metal movements, they either act at the plasma membrane (PM) to transport metals into cytoplasm or redistribute metals from intracellular compartments into the cytoplasm [6], [7], [8], [9]. Besides the above transporters families, novel transporters related to metal homeostasis are continuously under discovery, oligopeptide transporter (OPT) family is among those being focused recently [10], [11], [12]. OPT family is one of three families responsible for peptide transport; the other two are the ATP-binding cassette (ABC-type) transporters superfamily and the peptide transporter (PTR) superfamily [10]. Peptides, existing abundantly in xylem and phloem saps, are important for plant growth, development and signaling. Transport of peptides is taken as a more efficient means of nitrogen distribution than transport of individual amino acids, implying the important role of peptide transporters in long-distance transport of nutrients [10]. Besides the peptides, inorganic nutrients and transit metals have been reported as the substrates of peptide transporters in the recent studies. For intances, the ABC transporters AtMRP3 [13], AtATM3 [14] and AtPDR8 [15] are proposed to involve in heavy metal (Cd, Pb) resistance and movement.

The first OPT family is identified in the pathogenic yeast *Candida albicans* [16] and subsequently in *Schizosaccharomyces pombe* [17] and *Saccharomyces cerevisiae* [18]. Later, it is also found in bacteria, plants, and archaea [11], but still not in animals. OPT family is grouped into two distinct subfamilies, the yellow stripe (YS) clade and the peptide transport (PT) clade [11], [12]. The function of YS1-like (YSL) transporter on transportation of metal-complex has been documented well in *planta*. For example, YSLs mediate long-distance transport of specific Fe species [19], [20]. *ZmYS1* cloned from *Zea mays* is the founding member of YSL family, which functions in root Fe-phytosiderophore (Fe-PS) uptake from the soil [21]. *HvYS1* encoding a YSL transporter is a specific transporter for iron (III)-PS in barley roots [22]. Since non-Poaceae species do not synthesize phytosiderophores, the substrates of YSL transporters in dicotyledonous plant are suggested to be Fe(II)/Fe(III)-nicotianamine (Fe-NA) functioning in the long-distance transporting system [23], [24], [25]. NA is a chelator for several micronutrient metals and involves in the movement of micronutrients and heavy metals throughout the plant [26]. Three *YSL* genes cloned from *T. caerulescens* express at higher levels as compared with their *Arabidopsis thaliana* homologs with distinct patterns. TcYSL3 was further confirmed as a Fe/Ni-NA influx transporter [19]. Functional analysis in yeast demonstrated that TcYSL3 can transport both Ni-NA and Fe-NA complexes into yeast, indicating that TcYSL3 may be involved in the long-distance translocation of Ni in *T. caerulescens.*

In *Arabidopsis*, nine putative OPT orthologues (*AtOPT1* to *AtOPT9*) have been identified [27], [28]. OPT transporters predominantly recognize tetrapeptides and pentapeptides, thus they are also called peptide transporters, but this is challenged recently as *AtOPTs* have been proved to play a role in metal homeostasis and movements. Transcript of *AtOPT2* is induced in root by iron and zinc deficiency, and that of *AtOPT3* by iron, copper and manganese deficiency [28], [29]. AtOPT3 is further proved to involve in the movement of iron to the developing seeds [30]. Moreover, both AtOPT6 and AtOPT7 can transport cadmium or cadmium-glutathione conjugates when heterologously expressed in yeast [31], [32]. All these studies indicate that OPT family might play important roles in metal uptake and transportation.

In the present study, we isolated a member of the OPT family from a metal hyperaccumulator, *Thlaspi caerulescens*, named *TcOPT3.* We characterized its expression in different tissues, and conducted heterologous expression in yeast to investigate the role of *TcOPT3* in metal uptake. We demonstrated that TcOPT3 was involved in Fe/Zn/Cd/Cu transportation and it should be a very important component in the metal long-distance transport system, and contribute to heavy metal hyperaccumulation in the shoots.

## Results

### Identification of *TcOPT3* Gene

Degenerated primers were designed from the most conserved regions according to the *AtOPT3*, *BjGT1* and *ZmGT* mRNA sequence. We isolated the full-length cDNA from *Thlaspi caerulescens* by RACE PCR, and designated it as *TcOPT3* (Genebank accession no. HQ69984). The coding region of *TcOPT3* was 2211 bp, and a corresponding 737 amino-acid sequence was predicted.

**Figure 1:**
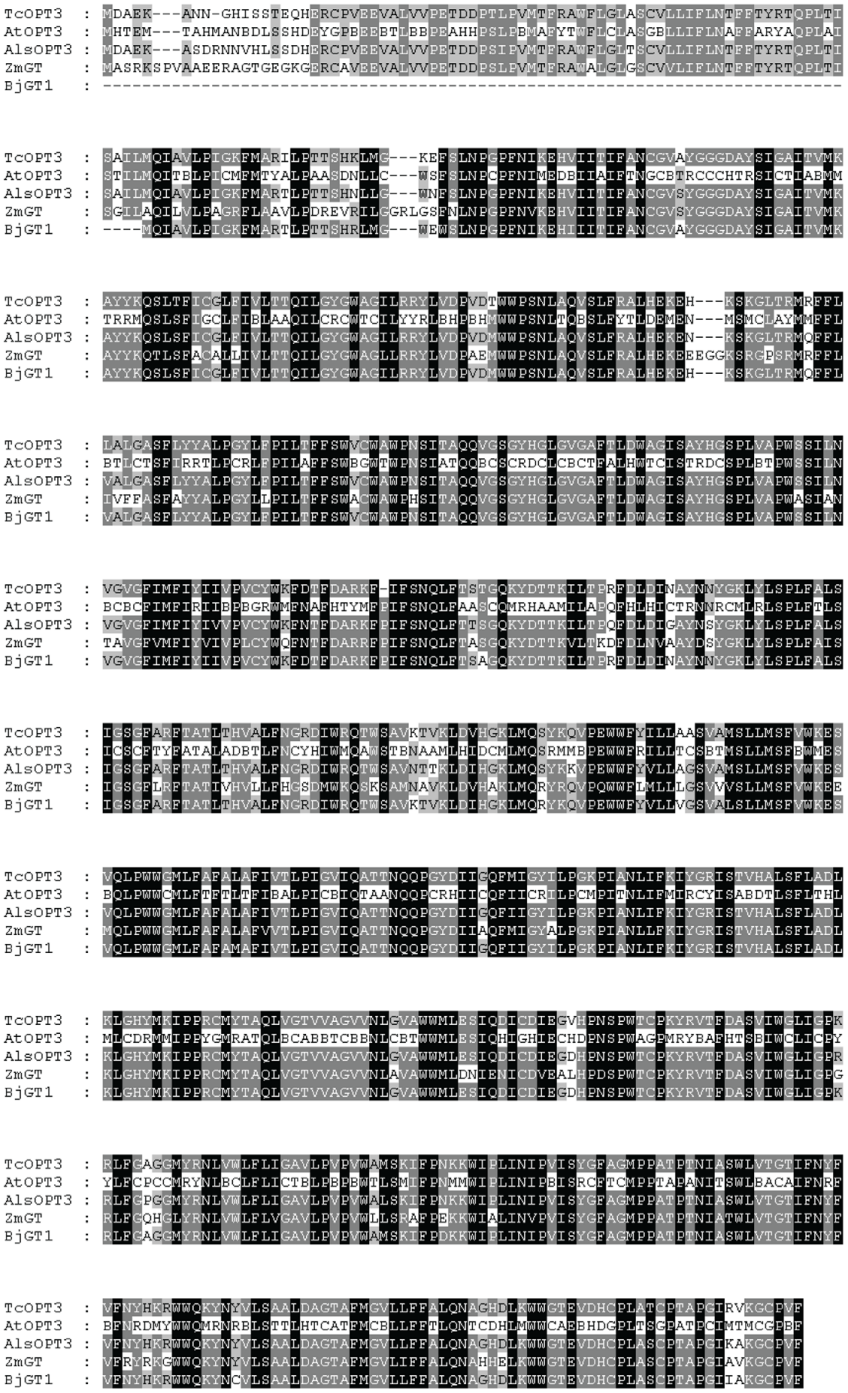
Sequence analysis of TcOPT3. Sequence alignment among the TcOPT3, AtOPT3 ATOPT3, ZmGT and BjGT1. CLUSTAL W (version 1.8) alignment of deduced amino acid sequences from the OPTs. Amino acids are numbered from the initiator ATG. Black-shaded areas represent the consensus, dark-gray-shaded areas represent identical amino acids, and light-gray-shaded areas represent similar amino acids. The putative transmembrane (TM) domains of the TcOPT3 were determined by the TMHMM algorithm. The predicted transmembrane membrane spanning domains are shown as lines above the sequences, and numbered TM I–TM XIV respectively. The bars under the sequence show the location of the two conserved motifs (NPG and KIPPR motifs). Tc, T. caerulescens; At, *Arabidopsis thaliana*; Als, *Arabidopsis lyrata subsp*.; Zm, Zea mays; Bj, Brassica juncea. Accession numbers are: HQ699884 (TcOPT3), NP_567493 (AtOPT3), XP_002868139 (AlsOPT3), ACL82964 (ZmGT), CAD91127.1 (BjGT1).

*TcOPT3* exhibited 79% identity with its homologous *AtOPT3*, and the deduced protein TcOPT3 showed 95% identity with AtOPT3 (Fig. 1). It contained 14 putative transmembrane domains (TM I-TM XIV, Fig. 1) and two highly conserved motifs (NPG motif and KIPPR motif, Fig. 1) of OPT family, thus TcOPT3 is likely to be localized to membrane and owns the structure of a transporter protein.

**Figure 2:**
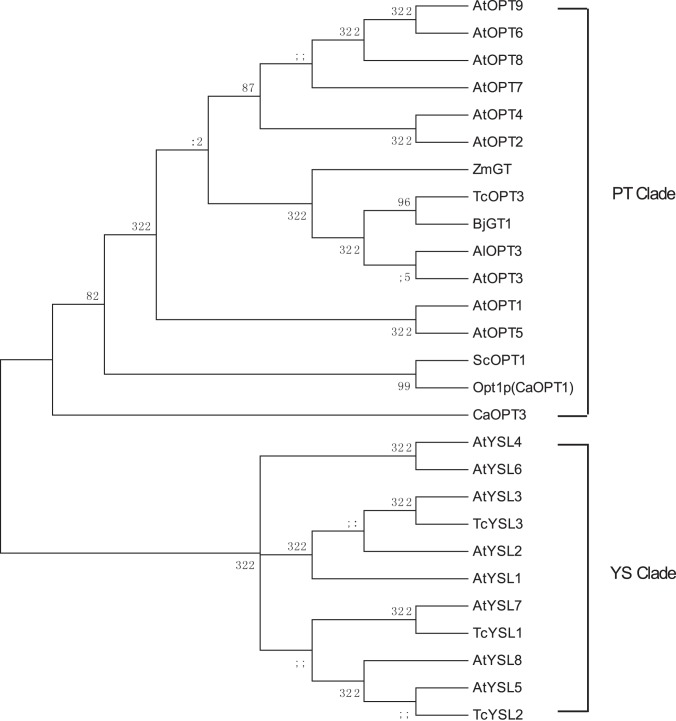
Phylogenetic tree of OPT gene transporters based on the amino acid sequences. Dendogram showing sequence comparisons of several known members of the PT family from different species. Analysis was performed using the CLUSTAL X method in MEGA (4.0) using Neighbor-Joining method (Tamura K et al., 2007). Accession numbers are as follows: AtOPT1, NP_200404.1 GI: 15241078; AtOPT2, NP_172464.1 GI15218331; AtOPT3, NP_567493.5 GI:240255930; AtOPT4, NP_201246.1 GI:15237689; AtOPT5, NP_194389.1 GI:15236800; AtOPT6, NP_194503.1 GI:15234254; AtOPT7, NP_192815.1 GI:15236912; AtOPT8, NP_564525.1 GI:18402162; AtOPT9, NP_200163.1 GI:15238761; AlOPT3, XP_002868139.1 GI:297800510; AtYSL1, NP_567694.2 GI:79484897; AtYSL2, NP_197826.2 GI:79518939; AtYSL3, NP_200167.2 GI:145359208; AtYSL4, NP_198916.2 GI:42568235; AtYSL5, NP_566584.1 GI:18401590; AtYSL6, NP_566806.1 GI:18405202; AtYSL7, NP_176750.1 GI:15218799; AtYSL8, NP_564525.1 GI:18402162; TcYSL1, ABB76761.1 GI:82468791; TcYSL2, ABB76762.1 GI:82468793; TcYSL3, ABB76763.1 GI:82468795; CaOPT1, AAB69628.1 GI:2367386; CaOPT3, ABD17824.1 GI:87045965; ScOPT1, NP_012323.1 GI:6322249; ZmGT, ACL82964.1 GI:220901863 BjGT1, CAD91127.1 GI:30722286.

From the rooted phylogenetic tree to compare TcOPTs with OPTs and YSLs in *A. thaliana* and the related species. We can see that TcOPT3 belongs to the PT clade, moreover, it clusters together in one branch with the OPTs from *T.caerulescens*, *B. juncea, A. lyrata* and *A. thaliana* and is most closely related to BjGT1 (Fig. 2).

### Tissue- and Organ- Specific Analysis of *TcOPT3*

**Figure 3:**
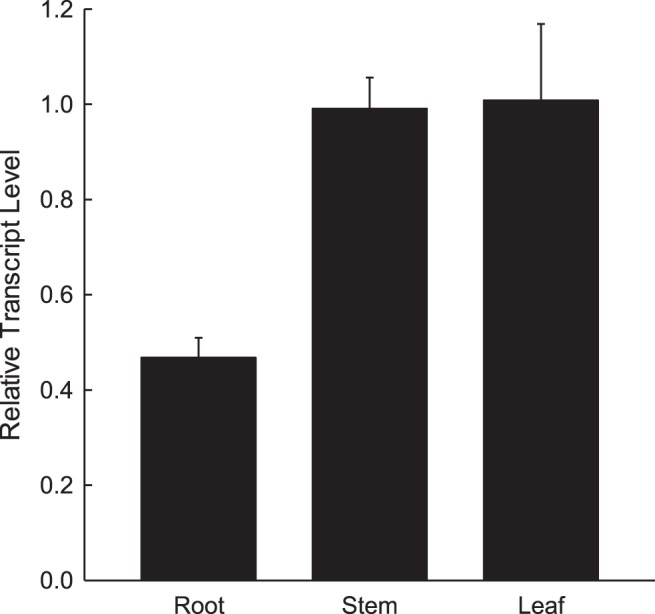
Tissue-specific analysis of the TcOPT3. Real-time RT-PCR expression analysis of the *TcOPT3* gene expression in roots (R), leaves (L) and stems (S). The ΔCp values were calculated as follows: CP of target gene (*TcOPT3*) – CP of constitutive control gene (*ubiquitin-conjugating enzyme*), where the CP value is the fractional cycle number of crossing point (CP). The ΔCP values represent the mean of three technical replicates (±SD) of one experiment representative of three independent experiments. Relative transcript levels (RTL) were calculated as follows: RTL = 2^−ΔCP^.

The expression of TcOPT3 was investigated by real-time RT-PCR, where ubiquitin-conjugating enzyme gene (*UBQ10*) was used as a control. The transcript level of TcOPT3 mRNA was 2-fold more in the leaf and stem tissues than that in the root (Fig. 3).

**Figure 4:**
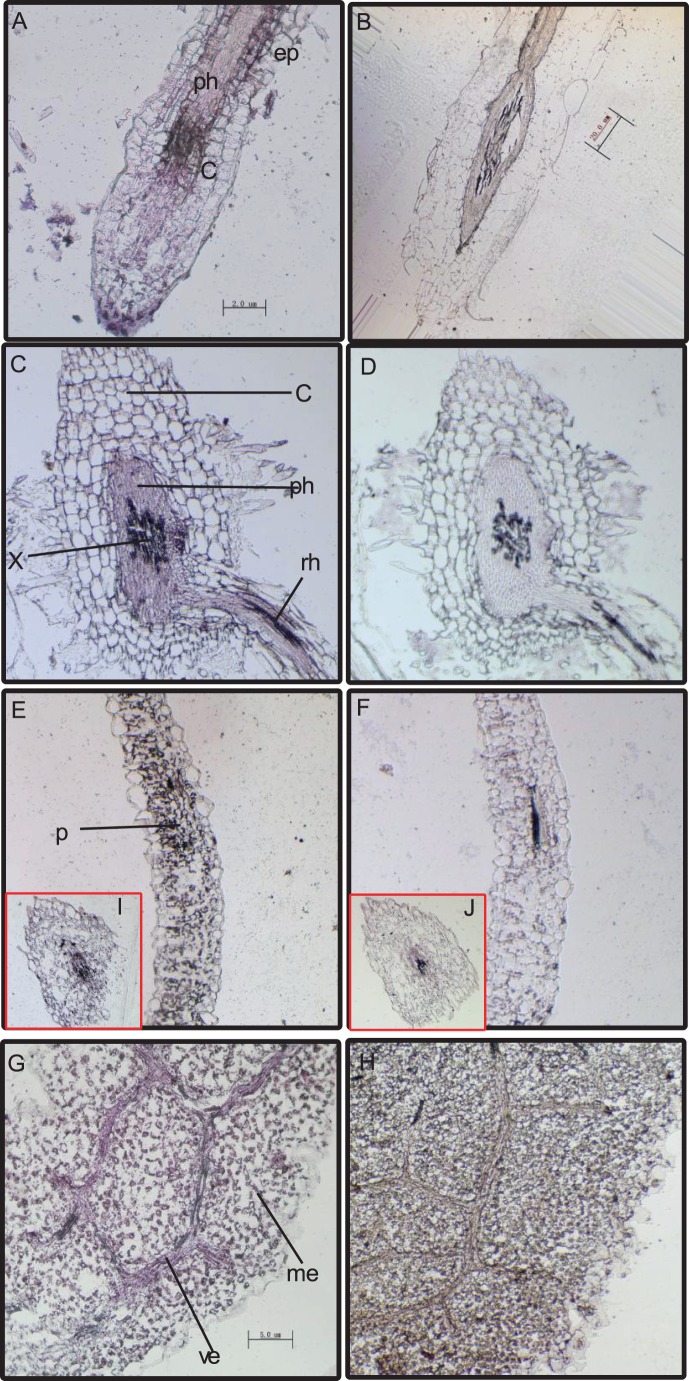
Localization of TcOPT3 expression by *in situ* hybridization. (A,C,E,G,I) represents hybridization with *TcOPT3* antisense probe. (B,D,F,H,J) shows hybridization with the sense probe (negative control). *In situ* hybridization of the sense and antisense *TcOPT3* probes to sections of *Thlaspi caerulescens* root tissues (A) to (D), stem tissues(E,F,I,J) and leaf tissues(G,H). Abbreviation: c, cortex; ep, epidermis; p, pericycle; ph, phloem; rh, root hair; x, xylem; cb, cambium; ve, vein; me, mesophyll; vc, vascular cambium.

To further identify the spatiotemporal expression of TcOPT3 *in vivo*, we performed *in situ* hybridization on the longitudinal and transverse sections of root, stem and leaf using DIG-labeled TcOPT3 antisense and sense RNA as probes (Fig. 4). Sense-oriented probe was used as the negative control (Fig. 4 B, D, F, H, J). In roots, more intense signals were detected in the pericycle and the vascular tissues, including xylem and phloem (Fig. 4A). This pattern of localization was further confirmed in the cross-section (Fig. 4C). In the stem, *TcOPT3* transcripts were strong in almost all the central cylinder cells (Fig. 4E, I). Additionally, in the leaf, extremely strong signals were detected in the vein of the mesophyll (Fig. 4G). All these suggest that as a transporter protein, TcOPT3 most probably plays a role in the long-distance transporting system.

### *TcOPT3* Expression in Response to Metal Deficiency

**Figure 5:**
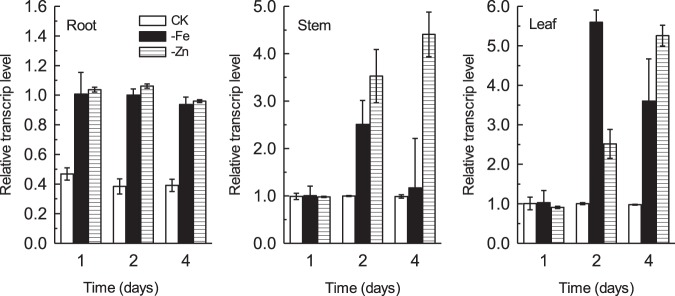
Effect of element deficiency on mRNA expression of the TcOPT3 gene. Real-time RT-PCR expression analysis of the *TcOPT3* gene expression in roots (R), leaves (L) and stems (S) with the treatment of Fe or Zn deficient for 1, 2, 4 days. The ΔCp values were calculated as follows: CP of target gene (*TcOPT3*) – CP of constitutive control gene (*ubiquitin-conjugating enzyme*), where the CP value is the fractional cycle number of crossing point (CP). The ΔCP values represent the mean of three technical replicates (±SD) of one experiment representative of three independent experiments. Relative transcript levels (RTL) were calculated as follows: RTL = 2^−ΔCP^.

To see whether the gene responses to metal deficiency, we measured the expression of *TcOPT3* in root, stem and leaf tissues (Fig. 5) and found it was also highly induced by Fe and Zn deficiency, especially in the stem and leaf. But the expression of *TcOPT3* responded to Fe and Zn deficiency faster in the root as the enhancement was detected in 1-day treatment, while 2-day in the aerial parts.

### Sub-Cellular Localization of the TcOPT3 Protein

**Figure 6:**
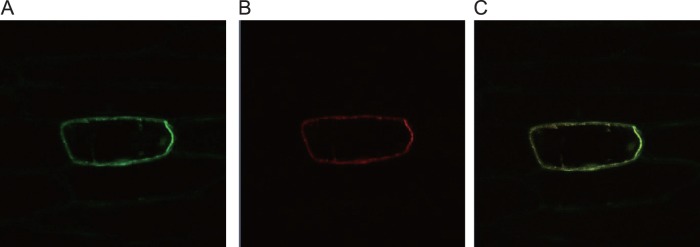
Sub-cellular localization of TcOPT3 protein. Onion epidermal cells transiently co-transformed with TcOPT3::GFP and pm-rk (Plasma membrane marker). (A) Fluorescence image of epidermal cell expressing the p35S::EGFP fusion protein. (B) Fluorescence image of epidermal cell expressing the pm-rk. (C) Merged fluorescence image of epidermal cell expressing the p35S-TcOPT3::EGFP fusion protein and pm-rk marker.

To investigate the sub-cellular localization, TcOPT3 fused with a green fluorescent protein (GFP) under the control of the cauliflower mosaic virus 35 S promoter and pm-rk (Plasma membrane marker) were co-expressed in the onion epidermis cells. The confocal microscopy observation showed that the green fluorescence and red pm-rk were both confined to the plasma membrane (Fig. 6) and can be merged perfectly, indicating that *TcOPT3* encoded a plasma membrane-localized protein.

### Functional Characterization of TcOPT3 by Heterologous Expression in Yeast

**Figure 7:**
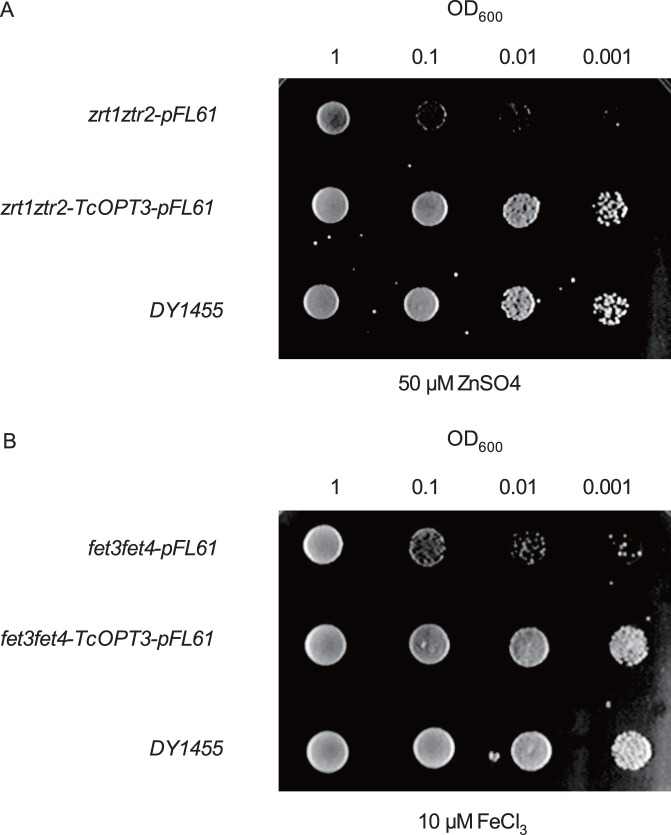
Complementation of the fet3fet4 and ZHY3 (zrt1ztr2) yeasts mutant by T. caerulescens cDNAs. Yeast strains defective in iron uptake (*fet3fet4*) and zinc uptake (*zrt1ztr2*) were transformed with pFL61 (empty vector) and pFL61-TcOPT3. Serial dilutions of yeast cells were dropped onto a low-zinc medium (LZM) supplemented with 50 µM ZnSO_4_ (A) and a low-iron medium (LIM) supplemented with 10 µM FeCl_3_ (B) assayed for growth on SD-ura plates. The entire experiment was performed twice.

To study the function of TcOPT3 in Fe and Zn transport, we tested whether expression of a *TcOPT3* cDNA could restore the growth of yeast double mutants *fet3fet4* (strain DEY1453) and *zrt1zrt2* (strain ZHY3), which can not grow on the Fe- and Zn-limited medium respectively [33], [34], [35]. As expected, expression of TcOPT3 complemented the growth of mutants *fet3fet4* and *zrt1zrt2* (Fig. 7) when both metal sources in the medium were provided at a low concentration (10 µM Fe^2+^ or 50 µM Zn^2+^), suggesting that TcOPT3 can transport both Fe^2+^ and Zn^2+^.

### TcOPT3 Contributes to Fe/Zn/Cu/Cd Accumulation

**Figure 8:**
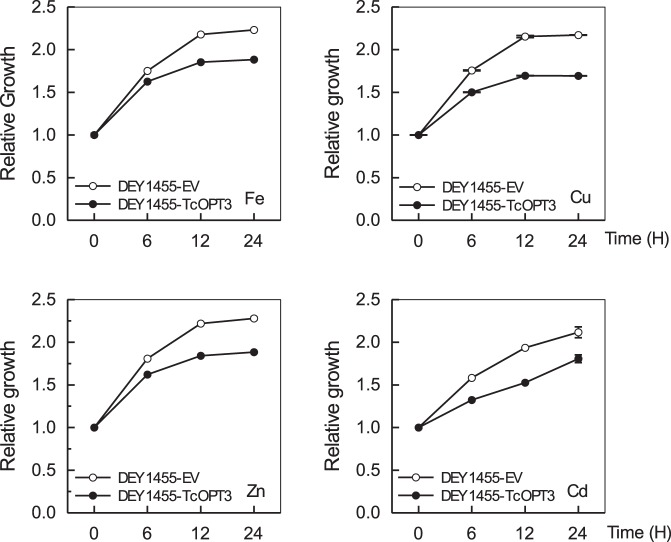
Growth of the wild-type (DY1455) and TcOPT3-transformed yeast cells under different metal supplies. Yeast cells were grown to an OD_600_ of 1.0, then supplemented with 50 µM FeCl_3_, 50 µM ZnSO_4_, 20 µM CdCl_2_ or 50 µM CuSO_4_ respectively. Data are the means ± SE per experiment (n = 3), P<0.05 by Student’s *t*-test.

**Figure 9:**
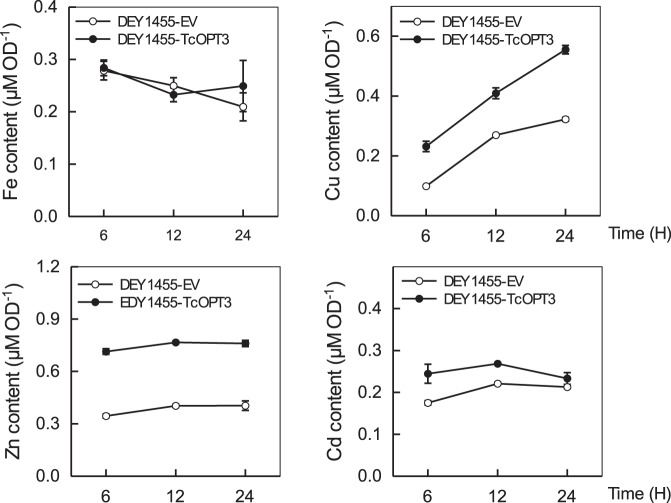
Heavy metal accumulation of wild-type (DY1455) and TcOPT3-transformed yeast cells. Zn, Fe, Ni, Cd and Cu accumulation in yeast transformants. Metal accumulation was conducted in liquid SD media supplemented with 50 µM FeCl_3_, 50 µM ZnSO_4_, 20 µM CdCl_2_, or 50 µM CuSO_4_ respectively. Data are the means ± SE per experiment (n = 3), P<0.05 by Student’s *t*-test.

The role of TcOPT3 in the metal accumulation was further investigated by expressing TcOPT3 and empty vector in the wild-type yeast line (stain DY1455) cultured in the solutions containing 50 µM FeCl_3_, 50 µM ZnSO_4_, 50 µM CuSO_4_ or 20 µM CdSO_4_, respectively. Compared with those empty vector-expressing strains, the TcOPT3-expressing trains grew worse (Fig. 8), meanwhile the corresponding contents of these metal ions were significantly higher as well, suggesting that TcOPT3 can transport Fe/Zn/Cd/Cu into yeast cells (Fig. 9).

## Discussion

The Oligopeptide transporters (OPTs) were initially characterized as small-peptide transporters. However, this has been challenged by their new functions in metal trafficking recently. The first report is from Wintz et al who found that heterologous expression of *AtOPT3* in yeast can transport Cu, Mn and Fe [28]. Later, AtOPT6 and AtOPT7 were also proved to transport Cd or Cd-glutathione chelate in yeast [31]. Recently, AtOPT3 was further demonstrated to be involved in Fe homeostasis [30]. It was showed that heterologously expressed AtOPT3 increased Cd sensitivity of *S. cerevisiae opt2* mutants and contributed to Cd accumulation [36]. All the above reports indicate the potential role of OPTs in transit metal transportantion. *Thlaspi caerulescens,* a Cd/Zn/Ni hyperaccumulator, ecotype of Ganges, from southern France, can accumulate 10 000 mg Cd kg^−1^ at shoot dry weight base [3], [37]. In this study, we cloned and elucidated the function of a novel member of OPT family, *TcOPT3* from *T. caerulescens*.

### Role of TcOPT3 in Metal Transport

AtOPT3 has been proved to play a role in Fe homeostasis in *A. thaliana* [30]. Here, we also found that heterologous expression of TcOPT3 in *Saccharomyces cerevisiae* rescued the growth of Fe-depleted mutant *fet3fet4* (Fig. 7), suggesting that TcOPT3 can transport Fe too. As *T. caerulescens* is also a Zn hyperaccumulator, we tested the possible role of TcOPT3 in transporting Zn. As expected, TcOPT3 restored the growth of Zn-uptake-defective mutant *zrt1zrt2.* Furthermore, heterologous expression of *TcOPT3* negatively affected the growth of *S. cerevisiae* when treated with elevated Fe, Zn, Cd, or Cu (Fig. 8) as more Fe, Zn, Cd or Cu was accumulated in the transformed yeast (Fig. 9), suggesting a broader substrates specificity for *TcOPT3*. The broad substrate specificity of cytoplasmic transition metal importers may ensure their universal potential to fulfill different developmental stages, without perpetuating the consequences of limited metal transporter specificity throughout the plant [7], while the specificity of transition metal export from cytoplasm may serve to establish specificity through the differential storage of transition metals in specific tissues of cell types. Therefore, the specificity of the efflux transporters appears to be more pounced than that of the influx transporters. For example, the P_1B_-type ATPases HMA3 from *T.caerulescens* showed high specificity for Cd, that serves to efflux Cd into vacoule [38]. As *T. caerulescens* can also accumulate Ni, we tested the possibility of transporting nickel (Ni), and found that altough the growth was negatively affected by Ni addtion (Fig. S1), the content in the yeast did not change much (Fig. S2), suggeting that TcOPT3 does not transport Ni, and there must be other transporters responsible for Ni hyperaccumulation, such as TcYSL3 [19]. Futhermore, we still found that TcOPT3 did not transport Pb into yeast too (Fig. S1, S2).

Roles of membrane transporters that are responsible for hyperaccumulation include uptake, efflux, translocation and sequestration of transit metals. Metal transporters herein identified include ZIP (ZRT/IRT like Protein), Nramp (Natural Resistance and Macrophage Protein), MTP (Metal Resistance Protein), and HMA (Heavy metal/CPX-type ATPases) families in *A.thaliana* and hyeperaccmulators such as *T. caerulescens* and *A. halleri* [4], [38], [39], [40], [41]. Transporters of the HMA family and the MTP family that are involved in metal efflux from the cytoplasm, either by movement across the plasma membrane (PM) or into organelles, whereas metal uptake transporters include the NRAMP family and the ZIP families that are transporters either act at the PM to move metals into the cytoplasm or remobilize metals from intracellular compartments into the cytoplasm [6]. In this study, heterologous expression of *TcOPT3* in *S. cerevisiae* increased the sensitivity of yeast by accumulating more metals, indicating that TcOPT3 function as an uptake transporter for heavy metals (Fig. 8, 9).

### Potential Role of *TcOPT3* in Plant Long-distance Metal Transport and Hyperaccumulation

In this study, qRT-PCR results showed that *TcOPT3* was preferentially expressed in the aerial parts (stem and leaf) than roots both under normal condition or Fe/Zn deficient conditions (Fig. 3, 5). While in Arabidopsis, *AtOPT3* expressed stronger in flower, leaf and root, but relatively lower in the stem [42]. It may be due to the different growth stage tested, but most probably, the different expression patterns between *AtOPT3* and *TcOPT3* may suggest their different roles in trafficking heavy metals within plants. It was reported that reduced expression of AtOPT3 in the Arabidopsis mutant *opt3-2* resulted in the accumulation of very high levels of Fe in tissues except seeds [30]. Recently, it was showed that heterologously expressed AtOPT3 increased Cd sensitivity of *S. cerevisiae opt2* mutants and contributed to Cd accumulation [36]. Consistent with the role of OPT3 in Cd detoxification, the early-stage seedlings of *A. thaliana opt3-3* knockdown allele were extremely sensitive to Cd. In contrast, leaves of hydroponically-grown *opt3-3* mature plants were more tolerant to Cd compared to the wild type [36]. We also found that heterologously expressed TcOPT3 increased heavy metal (Cu/Cd) sensitivity of *S. cerevisiae* and contributed to metal accumulation, suggesting a similar role of TcOPT3 in heavy metal trafficking in plant. However, little evidence from overexpression OPTs in metal distribution is reported to date. Given the different expression patterns between *TcOPT3* and *AtOPT3*, it is interesting to investigate the potential role of TcOPT3 in hyperaccmulation in *T. caerulescens*. The tissue specific overexperssion of TcOPT3 is highly desirable in order to verify its potential role in creating metal hyperaccumulators for the purpose of phytoremediation of heavy metal polluted environments in future.

As proposed by Milner and Kochian [4], Zn hyperaccumulation of *T.caerulescens* appears to include at least five physiological events: (1) Increased Zn^2+^ influx across the root-cell PM; (2) Reduced Zn sequestration in the root-cell vacuole; (3) Increased Zn transport into the xylem and via the xylem to the shoot; (4) Increased Zn^2+^ influx into leaf mesophyll cells; and (5) Zn and Cd stored primarily in leaf epidermal cells. *In situ* hybridization results showed that, *TcOPT3* was highly expression in vascular cells both in roots and shoots (Fig. 4). This expression pattern is similar to the previous report on the expression of AtOPT3 in *A. thaliana*, which is also highly expressed in the vascular tissues both in light-grown seedlings and adult plants [42]. As efficient translocation of metals from root to shoot is one of the most important hallmarks of hyperaccumulators. Thus, efficient transporters expressed in loading and unloading tissues are fundamental for hyperaccumulation. For example, HMA4 is characterized metal transporter primarily localized in the root stele. It plays a critical role in loading heavy metals into xylem for long-distance transportation from root to shoot not only in *T.caerulescens* but also in another hyperaccumulating plant *A.halleri* [43], [44], [45]. TcHMA3, another member of HMA family was proved to be a tonoplast-localized transporter highly specific for Cd, which is responsible for sequestration of Cd into the leaf vacuoles, and that a higher expression of this gene is required for Cd hypertolerance in the Cd-hyperaccumulating ecotype of *T. caerulescens* [38]. Uptake transporters responsible for hyperaccumulation, TcIRT1, TcIRT2, TcZNT1 and TcZNT5 of ZIP families in *T.caerulescens*, for instance, were expressed only in roots but not in leaves [46]. The yellow-stripe 1-like (YSL) subfamily is included in the OPT superfamily, some of which was proved to be involved in loading and unloading of nicotianamine-metal chelates from the vascular tissues. TcYSLs of YSL transporters from *T. caerulescens*, especially for TcYSL3 and TcYSL7, were expressed in xylem parenchyma and phloem [19]. Furthermore, TcYSL3 was shown to transport Ni-NA chelates. Here we demonstrated that TcOPT3 is a plasma membrane-localized protein, and can transport metals into yeast cells (Fig. 6, 8). TcOPT3 was expressed primarily in shoots, especially under nutrient deficiency, indicating their specificity in metal uptake and transport in shoots (Fig. 3, 5). As it is highly and constructively expressed in vascular system, including xylem, phloem and vein, it is reasonable to assume that it plays important roles in unloading of nutrients and heavy metals in those tissues, thus consists of an important component in the long-distance transportation system. However, it needs further investigation.

In this study, ecotype Ganges instead of ecotype Prayon was studied. Considered that heavy metal hyperaccumulation varies greatly among different ecotypes, it is interesting to further confirm the expression and functional analysis of TcOPT3 in two contrasting ecotypes of the hyperaccumulator *Thlaspi caerulescens*. We have analyzed the amino acids sequence and expression of TcOPT3-p preliminarily; however, there is no significant difference between TcOPT3-g and TcOPT3-p expression pattern. Further studies need to be conducted in the future.

In conclusion, we demonstrated that TcOPT3 in a metal hyperaccumulator, *Thlaspi caerulescens*, was an Fe/Zn/Cu/Cd influx transporter with non-specificity substrate. This is the first report showing that *TcOPTs* gene may be involved in metal long-distance transport systems that contribute to heavy metal hyperaccumulation.

## Materials and Methods

### Plant Growth

Seeds of *Thlaspi caerulescens J. & C. Presl*, ‘Ganges’ ecotype, were surface sterilized by 75% alcohol and 10% NaClO_4_, then stored at 4°C for 3 days. Seeds were germinated on agar with modified MS medium (with addition 270 µM ZnSO_4_ in Murashige and Skoog, Sigma, U.S.) for two weeks at 14-h/25°C d and a 10-h/18°C night regime, a light intensity of 100 µmol photons m^−2^ s^−1^. Then the seedlings were transferred to modified Hoagland nutrient solution (2 mM Ca(NO_3_)_2_, 0.1 mM KH_2_PO_4_, 0.5 mM MgSO_4_, 0.1 mM KCl, 0.7 mM K_2_SO_4_, 0.1 mM Fe-EDTA, 10 µM H_3_BO_3_, 0.5 µM MnSO_4_, 10 µM ZnSO_4_, 0.2 µM CuSO_4_ and 0.01 µM (NH_4_)_6_Mo_7_O_2_, pH 5.5).

### Gene and cDNA Cloning and Sequencing

Total RNA was extracted from *T. caerulescens* roots or leaves by the Trizol reagent (Invitrogen, USA) and purified by DNase I (RNase Free) Kit (TaKaRa, China). First strand cDNA of *T. caerulescens* synthesis was performed using M-MLV reverse transcriptase (TaKaRa, China) and PCR-amplified with degenerated primers designed from conserved *AtOPT3* sequence. One fragment was cloned into the pMD19-T Vector (TaKaRa, China), sequenced and identified as *AtOPT3* orthologs by BLAST using the NCBI database. RACE PCR (5′ and 3′) were performed in order to obtain full-length sequences from uncomplete cDNA fragments (see Table 1 for primer sequences). The 5′ and 3′ regions of cDNA fragments were cloned with a SMART™ RACE cDNA Amplification Kit (Clontech, TaKaRa Bio, USA) according to the manufacturer’s protocol. The PCR products were subcloned to pMD19-T Vector and sequenced. By sequencing alignment with its *AtOPT3* ortholog from *A. thaliana*, it was observed that the 5′ region of the isolated *TcOPT3* cDNA contained the first initiating ATG codon and the 3′ region containing the stop codon and the 3′ UTR. The gene we cloned has been deposited in Genbank with the accession number of HQ699884.

**Table 1:** Primers used for PCR amplification of in TcOPT3 cDNAs, 5′ and 3′ RACE, qRealTime-PCR, and plasmid constructions in order of their first mention in “Materials and Methods”.

Purpose	Name	Sequence (5′-3′)
RACE PCR	TcOPT3-GSP-F	CAGTAACAGACAGGGACGATG
	TcOPT3-GSP-R	CAGCAGGTTGGCTCAGGGTA
	TcOPT3-5′R-1-R	ACCTGCTGAGCCGTGATGCTATTA
	TcOPT3-3′R-1-F	ACATCATCGTCCCTGTCTGTTACTG
	TcOPT3-5′R-2-R	CGACGATTGAGTGCGAAAAGAG
	TcOPT3-3′R-2-F	CTTCAAATCTCGCTCAAGTTTCTCT
Quantified-RealTime PCR	qRT-TcOPT3-F	CCGAGCAGCACGAGCGTTGT
	qRT-TcOPT3-R	GCGGTTGCGTGCGGTATGTG
	TcUBQ-F	GGAGCCCCGCTTGGAC
	TcUBQ-R	CGGCGAGGCGAGTGTA
in situ hybridization	TcOPT3-Probe-S	CCGGAATTCGGTTATCTCCTACGGCTTTG
	TcOPT3-Probe-A	CCCAAGCTTTAGGGCAAGCAGTGGTATCAA
Plasmid of GFP fusion	TcOPT3-EGFP-F	AAAAGTACTATGGACGCAGAGAAGGCTAACAAT
	TcOPT3-EGFP-R	TCCCCCGGGGAAAACAGGACAGCCTTTAACTCT
Plasmid of yeast transformation	TcOPT3-pFL61-F	ATAAGAATGCGGCCGCATGGACGCAGAGAAGGCTAA
	TcOPT3-pFL61-R	ATAAGAATGCGGCCGCAATCTTAGAAAACAGGACAGC

F, Forword; R, Reverse. S, Sense; A, Anti-sense.

### Sequence Comparisons

The predicted amino-acid sequence from TcOPT3 cDNA and other selected OPT protein sequences were aligned using the CLUSTAL W program, version 1.8 (Thompson et al., 1994). The putative trans-membrane domains were predicted by the TMHMM (version 2.0; http://www.cbs.dtu.dk/services/TMHMM/). The putative amino acid sequences were aligned with the program Clustal X Version 1.8 [47] and viewed by GeneDoc version 2.6 [48]. Phylogenetic trees were constructed with the neighbor-joining algorithm using the program with MEGA 4 (http://www.megasoftware.net) [49]. Hydrophilicity plots for TcOPT3 was generated based on the Kyte and Doolittle (1982) method using Protean sequence analysis software (DNASTAR) under default parameters.

### Quantitative PCR Analysis of TcOPT3 Expression

Total RNA was isolated from roots, stems and leaves of one-month-old *T. caerulescens* and treated by DNase I (RNase free) as described above. RT-PCR with oligo d(T)-anchor primer were performed with PrimeScript RT (Perfect Real Time) reagent kit (TaKaRa, China). For real-time RT-PCR, 2 µl of the diluted (1∶5) cDNA products were used as templates with 5 µl SYBR Pre mix (Takara, China), 0.5 µl primers (10 µM each) and 2.5 µl water. Calculation of the ΔCp values was performed as described [50]. Primers were list in Table 1.

### *In Situ* Hybridization

One-month-old seedlings grown on hydroponic culture were obtained. Plant materials were fixed in potassium phosphate buffer (0.1 M, pH 7.4) containing 4% paraformaldehyde overnight at 4°C, then dehydrated in ethanol series, and embedded in paraffin. A gene-specific fragment containing the 416 bp fragment cross 3′UTR of *TcOPT3* was amplified by PCR (primers were shown in Table 1) and cloned into pSPT19 vectors. Sense and antisense probes were synthesized using SP6 and T7 primers with DIG RNA Labeling kit according to the manufacturer’s instructions (Roche, USA). *In-situ* hybridization was performed as described previously [19], [51].

### GFP Fusion and Subcellular Localization

To construct the TcOPT3-GFP fusion protein, the ORF without the stop codon of *TcOPT3* cDNA fragment containing *SmaI* and *SacI* restriction sites (see Table 1 for primer sequences) was cloned into the modified pEGFP-N2 vector under the control of 35 S promoter. The final construct p35S::TcOPT3-EGFP and the pm-rk were transiently expressed in onion epidermal cells using a particle gun–mediated system (PDS-1000/He; Bio-Rad), where pm-rk was used as the plasma membrane-localizated maker [52]. Onion epidermal cells were bombarded with 1 µm gold particles coated with plasmid DNA and then incubated in the dark at 25°C for 20 h. Fluorescence was observed by confocal laser scanning microscopy (LSM700; Carl Zeiss).

### Yeast Complementation

For yeast transformation and functional complementation, the Saccharomyces cerevisiae (Meyen) E.C. Hansen knock-out strain fet3fet4 (DEY1453; MATa/MATa ade2/+ can1 his3 leu2 trp1 ura fet3-2::HIS3 fet4-1::LEU2) [33], zrt1zrt2 (ZHY3; MATa ade6 can1 his3 leu2 trp1 ura3 zrt1::LEU2 zrt2::HIS3) [34], [35]; and its parent strain DY1455 (MATα ade2–1oc can1 his3 leu2 trp1 ura3) were used, which is defective in low- and high-affinity iron or zinc uptake, respectively. The ORF of TcOPT3 was amplified by PCR with primers (see Table 1 for primer sequences), which was then cloned into the pGEM-T Easy (Promega) vector, digested with NotI, and subsequently cloned into yeast binary vector pFL61 [53] to form TcOPT3-pFL61. Constructs were sequenced to ensure the correct orientations of the inserts and correct sequences. fet3fet4 transformants were selected on SD medium lacking uracil (SD-ura) and supplemented with 10 µM FeCl_3_. zrt1zrt2 transformants were selected on SD-ura and supplemented with 50 µM ZnSO_4_.

For complement test, *fet3fet4* and *zrt1zrt2* transformants were grown overnight in liquid SD-ura and supplemented with 10 µM FeCl_3_ or 50 µM ZnSO_4_, respectively. Yeast cells were recovered by centrifugation, resuspended in SD-ura (without added Fe or Zn) at an OD_600_ of 1, 0.1, 0.01 and 0.001. Then 10 µL of each culture was spotted on SD-ura plates; plates were incubated at 30°C for 2 d and then photographed.

### Heavy Metal Tolerance in Yeast

Wild-type yeast DY1455 was transformed with TcOPT3-pFL61, or with pFL61, which served as a negative control. DY1455 transformants were grown on liquid SD-ura medium overnight at an OD_600_ of 1. After washed by CaCl_2_ and centrifugation, resuspended in SD-ura with 50 µM FeCl_3_, 50 µM ZnSO_4_, 20 µM CdCl_2_, or 50 µM CuSO_4_ were grown for another 0, 6, 12, 24 hours. After measured the OD values and washed by CaCl_2_ to remove adsorbed transit metals in apoplast cells, aliquots of yeast cells were taken for measurement of metal accumulation.

### Elemental Analysis

For metal accumulation, yeast cells were concentrated at 5,000 rpm for 5 min and washed by 0.5 mM CaCl_2_ for 5 min twice to remove adsorbed transit metals from the yeast cell walls. Then cell sediments were dried at 70°C for 2 days, and digested in HNO_3_. Metal concentration in the digested solution was determined by inductively coupled plasma-atomic emission spectrometry (ICP-AES; IRIS/AP optical emission spectrometer).

### Statistics

Data were statistically analyzed using analysis of variance (ANOVA) in Origin 8, and tested for significant (P≤0.05) treatment differences using Student’s *t*-test.

## Supporting Information

Figure S1 **Growth of the wild-type (DY1455) and TcOPT3-transformed yeast cells under different metal supplies.** Yeast cells were grown to an OD_600_ of 1.0, and then supplemented with 100 µM PbNO_3_ or 100 µM NiSO_4_ respectively. Data are the means ± SE per experiment (n = 3), P<0.05 by Student’s *t*-test.
(EPS)

Figure S2 **Heavy metal accumulation of wild-type (DY1455) and TcOPT3-transformed yeast cells.** Pb and Ni accumulation in yeast transformants. Metal accumulation was conducted in liquid SD media supplemented with 100 µM PbNO_3_ or 100 µM NiSO_4_ respectively. Data are the means ± SE per experiment (n = 3), P<0.05 by Student’s *t*-test.
(EPS)
